# Evaluation of Osteogenic Potential of a Polysaccharide-Based Hydrogel Coating on Titanium

**DOI:** 10.7759/cureus.57785

**Published:** 2024-04-07

**Authors:** Rashmita Majhi, Tapan K Patro, Angurbala Dhal, Satish Kumar, Puspendu Guha, Luna Goswami, Chandan Goswami, Rakesh K Majhi, Lokanath Garhnayak

**Affiliations:** 1 Department of Prosthodontics and Crown & Bridge, SCB Dental College and Hospital, Cuttack, IND; 2 School of Biotechnology, Kalinga Institute of Industrial Technology, Bhubaneswar, IND; 3 School of Biological Sciences, National Institute of Science Education and Research, Bhubaneswar, IND; 4 Department of Experimental Condensed Matter Physics, Institute of Physics, Bhubaneswar, IND; 5 School of Chemical Technology, Kalinga Institute of Industrial Technology, Bhubaneswar, IND; 6 Department of Biological Sciences and Bioengineering,, Mehta Family Center for Engineering in Medicine, Indian Institute of Technology Kanpur, Kanpur, IND; 7 Center of Excellence for Cancer, Gangwal School of Medical Science and Technology, Kanpur, IND

**Keywords:** implant osseointegration, bone mineralization, functional biomaterials, dental implantology, bone repair

## Abstract

Introduction: Reducing the healing period after surgical placement of dental implants can facilitate the loading of dental prostheses.

Aim: The aim is to compare the osteogenic potential of unmodified titanium disks with titanium disks that were surface-modified or hydrogel-coated.

Materials and methodology: One hundred eight titanium disks (Ø6 × 2-mm) were divided into three groups: (1) unmodified titanium as control (Ti-C); (2) sandblasted and acid-etched (Ti-SLA), and (3) coated with tamarind kernel polysaccharide hydrogel grafted with acrylic acid (Ti-TKP-AA). The osteogenic potential and cytotoxic effect of various groups of titanium were compared using human osteoblasts Saos-2. The surface topography of the titanium disks and morphology of osteoblasts grown on disks were investigated by scanning electron microscopy (n = 3). Cell attachment to the disks and actin expression intensity were investigated by confocal imaging (n = 3). Cytotoxicity was quantified by cell viability assay (n = 9). Osteoblast maturation was determined by alkaline phosphatase assay (n = 9). Cell mineralization was quantified by Alizarin red staining (n = 9). One-way analysis of variance followed by Tukey's multiple comparisons test was used for intergroup comparisons (*α*= 0.05).

Results: The surface modifications on Ti-SLA and Ti-TKP-AA support better morphology and proliferation of osteoblasts than Ti-C (P< 0.001) and significantly higher levels of actin cytoskeleton accumulation (P< 0.0001). Ti-TKP-AA showed a significantly higher maturation rate than Ti-C (P< 0.001). Ti-TKP-AA showed > twofold increased mineralization than Ti-C and Ti-SLA (P< 0.001).

Conclusions: TKP-AA hydrogel-coated titanium promotes faster osteoblast proliferation, maturation, and mineralization than SLA-treated or untreated titanium. These advantages can be explored for achieving early osseointegration and prosthetic loading of titanium dental implants.

## Introduction

Dental implants are an established treatment modality for the functional and esthetic rehabilitation of missing teeth [1,2]. Materials for dental implants need to be stable, chemically inert, corrosion resistant, biocompatible, and should have osteogenic potential [2]. The most popular material for implants in dentistry is still commercially pure titanium (grades 1-4), primarily due to its exceptional mechanical strength, corrosion resistance, and biocompatibility [2-4]. Titanium forms a stable titanium oxide (TiO2) layer on the surface that is biocompatible and provides high corrosion resistance [5].

Early osseointegration of dental implants is necessary for their clinical success [6]. The material surface affects cell attachment, differentiation, and mineralization, which leads to osseointegration of the implant [7]. Various chemical and physical surface modifications are being developed to improve osseous healing [1]. An implant material's surface properties can be enhanced in two ways: first, by optimizing the micro-roughness through techniques like sandblasting, acid etching, TiO2 airborne particle abrasion, anodic oxidation, or hydrofluoric acid treatment; and second, by using bioactive coatings made of collagen, hydroxyapatite, or bioactive glass [2,8]. The sandblasted and acid-etched (SLA) surfaces have demonstrated enhanced bone apposition in histomorphometric studies and higher removal torque values in biomechanical testing [9].

Clinically, a healing time of three to six months is recommended before the definitive prosthetic restoration can be given [10]. Currently, research attempts are focused on achieving a shorter healing period for implants, in order to load them as soon as possible after surgery, without compromising the efficiency of osseointegration. This is more critical in the bones of inferior quality and posterior maxilla [11].

Tamarind kernel polysaccharide (TKP), a naturally occurring polysaccharide originating from plants, has demonstrated great biocompatibility and suitability for the growth of various non-osteogenic and osteogenic cells when grafted with hydrophilic acrylic acid (AA) through radical polymerization [12]. In an in vitro investigation, TKP-AA likewise demonstrated a high rate of mineralization and differentiation of Saos-2 osteoblasts without a requirement for any addition of exogenous growth factors [13]. TKP-AA has the potential to be used as a scaffold for diverse cell types and particularly for bone tissue engineering at low cost [12]. However, its efficacy on dental implants has not yet been studied. The present study aimed to examine and compare the biocompatibility and osteogenic potential of TKP-AA coating on titanium disks with unmodified titanium and SLA-treated titanium. The null hypothesis of this study was that: (1) There would be no significant difference in cytotoxic effect among the different titanium study groups; (2) there would be no significant difference in osteogenic potential among the different titanium study groups.

## Materials and methods

Preparation of titanium specimens

A total of 108 disk-shaped specimens measuring Ø6 × 2 mm were generated by using a computerized wire electrical discharge machine (CNC EDM machine; Ocean Technologies Co, Ltd) to mill grade IV titanium (Tremor alloys). Specimen disks were polished in a sequential manner with silicon carbide sheets with grit sizes 240, 600, and 1,200. The disks were cleaned for 10 min in an ultrasonic bath using a solution of 40% sodium hydroxide and 50% nitric acid to eliminate impurities and then rinsed with deionized water to achieve a neutral pH. Then, disks were immersed in 70% ethanol for 10 min to avoid microbial contamination and stored at room temperature [11,14]. The disks were divided into three groups: (1) control group (Ti-C) without any surface modifications; (2) sand-blasted & acid-etched titanium disks (Ti-SLA); (3) TKP with acrylic acid hydrogel-coated titanium disks (Ti-TKP-AA). For SLA treatment, cleaned titanium disks were subjected to airborne-particle abrasion with 0.25 mm grit alumina at 3-bar pressure until the surface reached a uniform gray tone, followed by acid etching in 37% hydrochloric acid (Sigma Aldrich) for three minutes. The disks were then rinsed in deionized water, neutralized in a 5% sodium bicarbonate solution, and then rinsed three times to five minutes each time in an ultrasonic bath [9,15]. TKP-based hydrogel was prepared as described before [12]. 20 µl TKP-AA hydrogel was coated by the drop coating method on the disks and the disks were air-dried. Each of these disks was UV sterilized for 90 minutes in a laminar airflow hood prior to cell culture [16].

Surface Morphology Analysis

Following the gold sputter coating process using a thermal evaporator, the surface topography of three disks (control and modified) was analyzed using a scanning electron microscope (Neon 40, Carl-Zeiss) to look for surface topography alterations (×3,000-100,000 magnification) [13].

Cell Culture

Adherent human osteoblast Saos-2 cells were regularly grown at 37°C in a humidified atmosphere of 5% CO2 in McCoy's 5A medium (Pan Biotech, Germany), supplemented with 10% fetal calf serum (Gibco), 50 IU penicillin/mL, and 50 µg/mL streptomycin (Himedia, Mumbai) [13].

Cell Morphology Analysis

10,000 cells were counted by hemocytometer and seeded on disks specimens (n = 3) placed in 48 well culture plates. The morphology of Saos-2 cells grown for 24 hours on each group specimen was examined by scanning electron microscope (Neon 40, Carl Zeiss) [13]. 

Cell Attachment Analysis

10,000 Saos-2 cells were seeded on disk specimens (n = 3) placed in 48 well culture plate. The Saos-2 cells attached to disks were fixed by using 2% Paraformaldehyde after 24 hrs and stained to reveal the polymerized filamentous actin cytoskeleton using Invitrogen's Alexa Fluor-488-labeled Phalloidin (1:200 dilution). Using a 63× objective, confocal pictures were captured using LSM-800 confocal microscope (Carl Zeiss) [13,17].

Cell Viability Analysis

Approximately, 10,000 Saos-2 cells were cultured on specimen disks (n = 9) for five days in a 24-well culture plate. Subsequently, the cells were incubated for four hours with 100 µL of 4,5-dimethylthiazol-2yl-2,5-diphenyltetrazolium bromide (MTT) reagent at 37°C. Following that, a buffer solution containing 50 mL of 0.02 M HCl, 50 mL of isopropanol, and 4 g of Nonidet P-40 detergent (Sigma Aldrich) was incubated for an hour at 37°C to dissolve the MTT formazan crystals. Using a microplate reader (Epoch2; BioTek), the optical density of the dissolved MTT formazan crystals was determined at 570 nm [13].

Cell Maturation Analysis

An alkaline phosphatase (ALP) assay was used to measure osteoblast maturation. A disk specimen (n = 9) was used to seed about 10,000 Saos-2 cells, which were then grown in an osteogenic medium containing McCoy's 5A, 10 mM β-Glycerophosphate, and 100 mM L-ascorbic acid (Sigma Aldrich). Cells cultured on the disks were lysed in cell lysis buffer (0.1 vol% Triton X-100) and rinsed with phosphate-buffered saline (PBS) after seven days. After that, 190 µL of p-nitrophenyl phosphate (Sigma Aldrich) was added, and the mixture was left to incubate in the dark for 30 minutes. By adding 100 µL of 3M sodium hydroxide solution, the process was stopped. Using a Varioskan LUX microplate plate reader (ThermoFisher Scientific), a spectrophotometric reading was obtained at 405 nm [13].

Cell Mineralization Analysis

Using alizarin red staining, the amount of calcium deposition in osteoblasts was assessed (n=9). Each disk containing 50,000 cells was cultured in a 48-well culture plate with mineralization induction media (McCoy's 5A media, which contained 10 mM β-Glycerophosphate, 100 mM L-Ascorbic acid, and 10 nM Dexamethasone) that was replaced every three days. Cells were fixed with 2% paraformaldehyde and rinsed with PBS after 21 days. Subsequently, 40 mM alizarin red solution (ARS) at pH 4.1 (Sigma Aldrich, Germany) was used to stain the cells. Using cetyl pyridinium chloride (CPC, MP Biomedicals) to remove calcium bound with ARS dye and a microplate reader (Epoch2; BioTek) to capture a spectrophotometric measurement at 550 nm, the amount of mineralization was measured [13].

Atomic Force Microscopy and Contact Angle Measurement

Microscopic measurements were conducted using Agilent 5500 AFM in soft tapping mode. Aluminum-coated silicon cantilevers from Nanosensors (PPP-NCHR) were used as AFM probes. The force constant and resonance frequency of cantilevers during the imaging were 30-34N/m and ≈300kHz, respectively. The contact angle was measured using DSA 25 Contact Angle Goniometer (Kruss Scientific) by dropping 2µL of MilliQ water onto the Titanium and TKP-AA coated Titanium disks, followed by videography for 3 min.

Ethics committee review

This study used human cell lines and not live animal or animal-derived primary cells. Hence it is exempt from review by the Institutional Review Board for Animal Ethics or Human Ethics.

Statistical analysis

Results from three to nine independent experiments were represented as mean ± standard deviation. Statistical analysis was performed by GraphPad Prism software (version 8.3.0). The conformity of the data to normal distribution was checked with the Shapiro-Wilk test (P > 0.05). One-way analysis of variance with Turkey’s multiple comparison post-test was performed to determine statistical significance between groups. The significance level was set to P < 0.05 [18].

## Results

**Figure 1:**
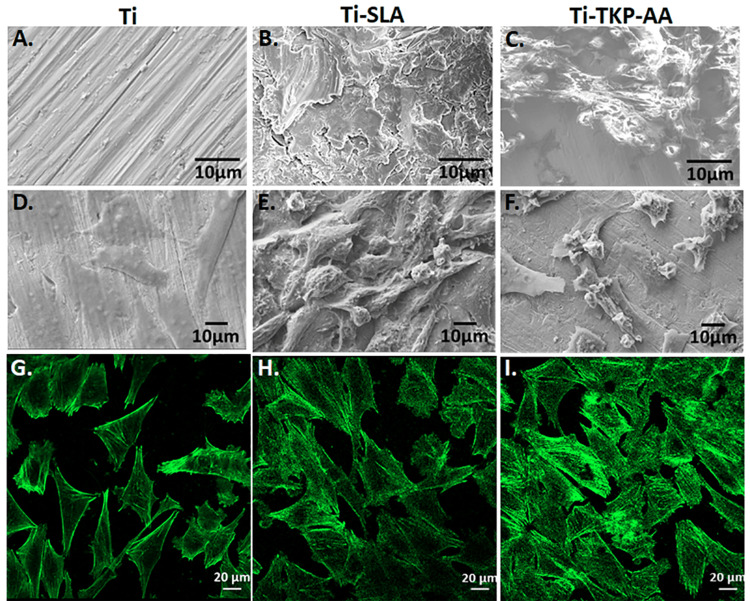
Effect of surface modification on osteoblast growth. Titanium disk surface topography is shown in Scanning Electron Microscopy pictures as follows: (A) unmodified, (B) treated with sand blasted and acid-etched (SLA), and (C) coated with tamarind kernel polysaccharide hydrogel grafted with acrylic acid (TKP-AA). photomicrographs of human osteoblasts Saos-2 cells attached on titanium disks after 24 hours of culture: (D) without surface modification, (E) treated with SLA, and (F) coated with TKP-AA hydrogel. Confocal pictures of Saos-2 cells grown on titanium disks for 24 hours, stained for filamentous actin (green) using Alexa 488-labelled Phalloidin: (G) unmodified surface, (H) treated with SLA, and (I) coated with TKP-AA hydrogel.

The surface topography of the disks by SEM revealed a uniform, smooth appearance on the surface of control titanium disks (Ti) (Figure 1A). SLA-treated titanium disk surface (Ti-SLA) revealed a rough surface with wavy structures (Figure 1B). Titanium disks coated with TKP-AA hydrogel (Ti-TKP-AA) had a uniform appearance and grain boundaries were seldom observed (Figure 1C).

The morphology of Saos-2 cells on titanium disks with modified surfaces and control was investigated using confocal and scanning electron microscopy. The surfaces of control Ti disks were uniformly covered with a monolayer of polygonal-shaped, flattened cells (Figure 1D). Cells cultured on Ti-SLA disks showed clusters of multilayered cells, with a 3D appearance but without much of cellular extensions (Figure 1E). Cells cultured on Ti-TKP-AA disks were more flattened and produced filopodia-like extensions. Clusters of multilayered cells were also observed in some areas (Figure 1F). This indicated that the surface treatments supported cell growth and the cells had different morphology based on surface modification present.

Through the use of confocal microscopy, the morphology of Saos-2 cells labeled for filamentous actin was further verified. Since filamentous actin is enriched in the sub-membranous region, hence we could also visualize the boundaries of the cells. Actin staining revealed that the osteoblast cells adhered and spread out well with many bundles of actin microfilaments on SLA-treated (Figure 1H), TKP-AA hydrogel-coated (Figure 1I) disks. In TKP-AA-coated disks, Saos-2 osteoblast cells were seen to be more densely packed (Figure 1I), suggesting a high rate of bone cell proliferation. Relatively fewer cells with thin actin microfilaments were visualized on the surfaces of control disks (Figure 1G). The control titanium disks also showed flattened cells, which accords well with the SEM analysis (Figure 1G).

**Figure 2:**
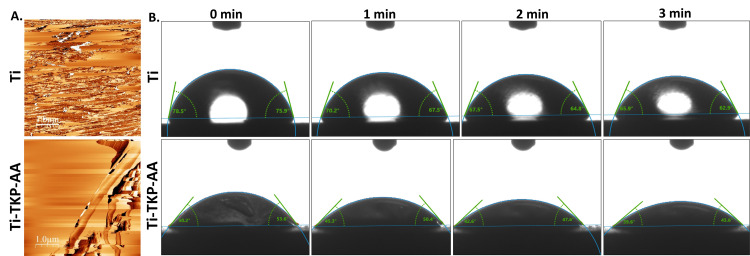
AFM and contact angle measurements. (A) Titanium disk surface topography of Ti and Ti-TKP-AA is shown as AFM images. (B) Contact angle measurements of Ti and Ti-TKP-AA are shown at 0, 1, 2, 3 min. Data are shown from three independent disks.

AFM analysis revealed a comparatively greater extent of smoothness on TKP-AA-coated Titanium disks as compared to Titanium disks (Figure 2A). The TKP-AA coating on Titanium disks increases the wettability of the disks, compared to titanium-only disks, as revealed by the smaller contact angles on TKP-AA-coated Titanium disks (Figure 2B, Table 1).

**Table 1:** Contact angle measurements. Contact angle of Ti and Ti-TKP-AA measured using Goniometer. Data are from two angles per disk and three disks in total. Mean and Standard deviation is shown. N=6.

	0 min	1 min	2 min	3 min
Ti	75.1 ± 9.6	67.1 ± 7.2	64.4 ± 6.7	61.7 ± 7.3
Ti-TKP	55.4 ± 14.4	48.6 ± 11.1	45.2 ± 9.5	41.8 ± 8.6

**Figure 3:**
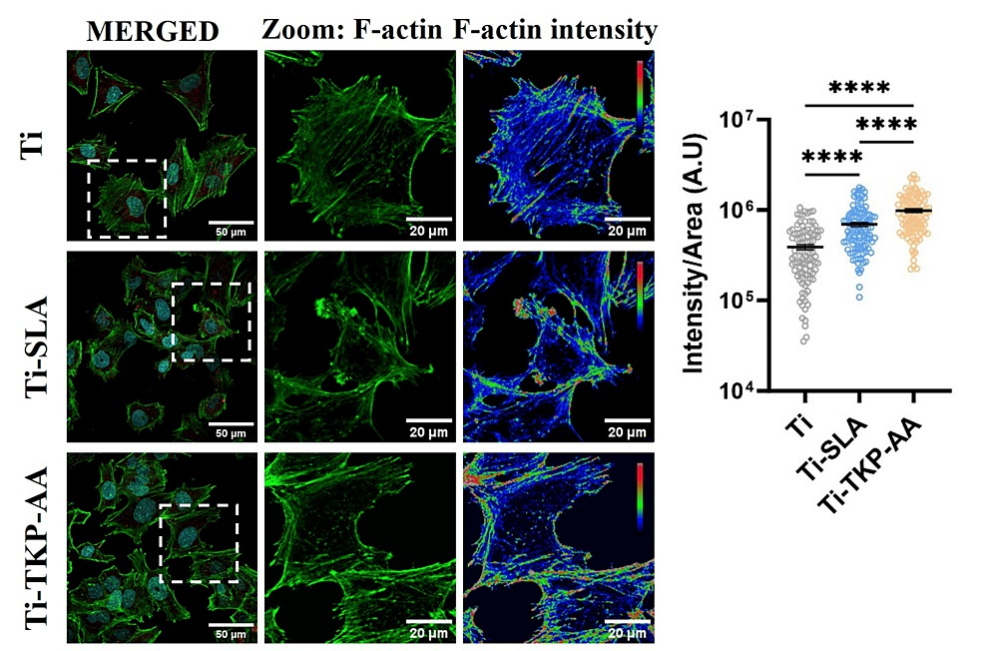
Effect of TKP-AA hydrogel on actin network of osteoblasts. Confocal images of Saos-2 cells grown on titanium disks for 24 hours without surface modification, with sand blasted and acid-etched (SLA) treatment and tamarind kernel polysaccharide hydrogel grafted with acrylic acid (TKP-AA) coating. The cells were stained for filamentous actin (green) using Alexa 488-labelled Phalloidin. In the magnified picture, the actin expression intensity is displayed as a pseudo-colored heatmap. Three separate experiments are represented by representative photographs. Graph represents Mean ± SD. n = 3, **** = P < 0.0001.

Quantification of actin intensity revealed significantly higher (P < 0.0001) actin levels in surface-modified titanium disks, with the highest filamentous actin levels in TKP-coated disks as compared to unmodified disks (Figure 3).

**Figure 4:**
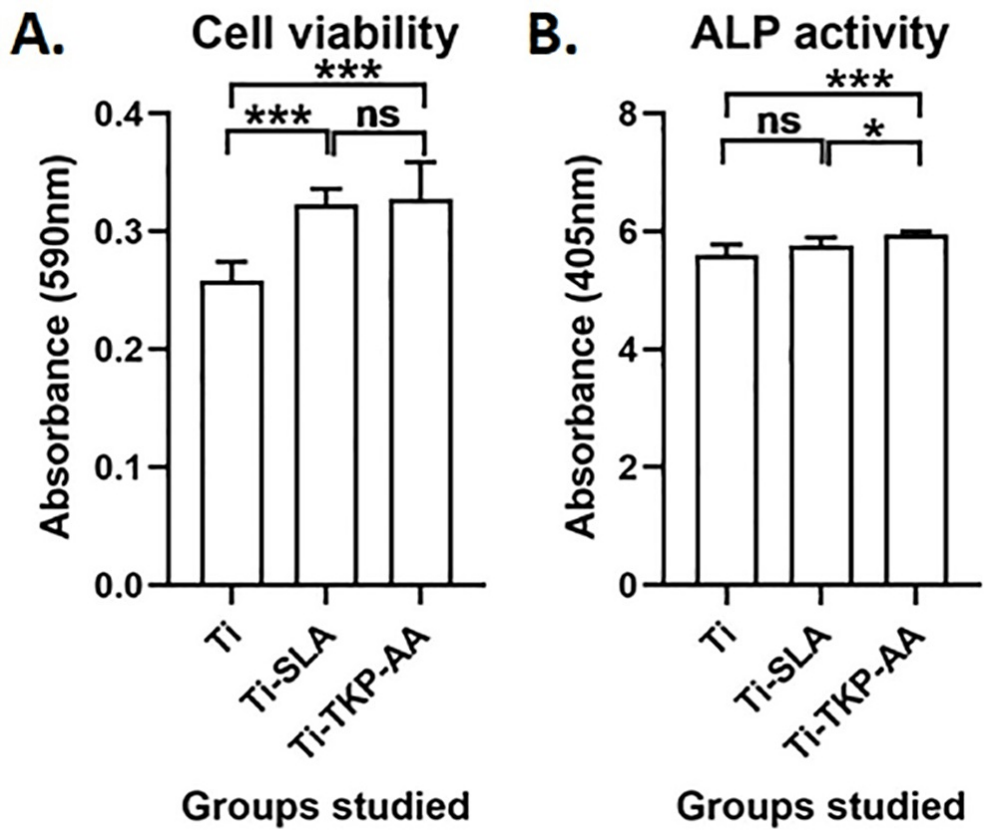
Effect of TKP-AA hydrogel on osteoblast proliferation and maturation. (A) MTT assay of Saos-2 cells cultured for five days showing cell viability of sand blasted and acid-etched (SLA) treated and tamarind kernel polysaccharide hydrogel grafted with acrylic acid (TKP-AA)-coated disks in comparison to only titanium. Higher cell viability than the only titanium control condition is indicative of higher proliferation rates supported by the surface modifications. (B) Alkaline Phosphatase assay using Saos-2 cells grown on control and surface modified titanium disks for seven days. Graphs represent Mean ± SD. n = 9, * = P <0.05, *** = P < 0.001.

The vitality and proliferation rates of Saos-2 cells cultured on control titanium and their surface changes were evaluated using the MTT test. The absorbance values verified that none of the treatments decreased the Saos-2 cells' viability (Figure 4A) demonstrating that each of the surface modifications had no cytotoxicity on osteoblasts. Interestingly, SLA treatment increased cell proliferation by 25% (P < 0.001) and hydrogel coating by 27% (P < 0.001) (Figure 4A). This indicated that these treatments significantly increased rates of bone cell proliferation.

Alkaline phosphatase activity is essential in osteoblasts to maintain their osteogenic properties and is a biomarker of differentiated osteoblasts. ALP assay revealed similar ALP activity in control titanium and SLA-treated titanium, hydrogel-coated titanium (Figure 4B). This indicated that titanium and the surface modifications equally supported osteoblast growth and the osteoblasts were healthy while growing on these surfaces.

**Figure 5:**
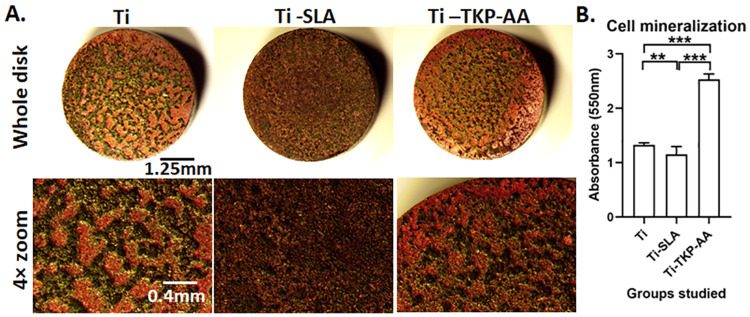
Effect of TKP-AA hydrogel on osteoblast mineralization. (A) Representative images depicting alizarin red stained mineralized nodules on Ti, sand blasted and acid-etched (Ti-SLA) and tamarind kernel polysaccharide hydrogel grafted with acrylic acid (Ti-TKP-AA) coated whole titanium disks and zoomed-up regions after 21 days of culture with Saos-2 osteoblasts. (B) Quantification of mineralization on control and surface modified titanium disks. Graph represents Mean ± SD. n = 9, ** = P < 0.01, *** = P < 0.001.

Bone cells deposit large quantities of calcium inside osteoblasts during the ossification and mineralization process. These calcium deposits can be stained by Alizarin Red. The titanium surface showed a high amount of calcium nodules, whereas, in SLA-treated titanium and TKP hydrogel-coated titanium, more dense nodules were formed. These results were further confirmed and quantified by dissolving the Alizarin red stain in 10% CPC solution and taking absorbance at 550 nm. The absorbance values indicated a nearly twofold increase in mineralization in TKP-AA hydrogel-coated disks (P < 0.001) (Figures 5A, 5B). This indicated that TKP-AA coating promotes more mineralization in osteoblasts.

## Discussion

There is a lot of research being done on dental implant surface modifications on CP titanium to get more bone volume and bone-to-implant contact. It has been demonstrated that improving the surface topography through airborne particle abrasion and acid etching or altering the surface's physicochemical characteristics through coating deposition will enhance the implant surfaces' osteoconductive and bioactive qualities [19]. The adsorption of proteins, the adherence of osteoblastic cells, and ultimately the rate of osseointegration are significantly influenced by surface characteristics in the nanometer range [6]. To provide titanium dental implants with a rough surface and enhance osseointegration, a number of techniques have been devised, including plasma spraying, anodization, ceramic particle blasting, and acid etching [6,9]. When employed as a blasting material, alumina (Al2O3) produces surface roughness that varies depending on the granulometry of the blasting media [6]. Presently, many of the titanium implants are grit blasted or acid etched (SLA) to roughen the surface and increase the area for bone contact [9,15]. Hence, SLA-treated titanium was used to compare its effectiveness to unmodified titanium disks.

Implant coating with polymeric materials like hydrogels can be considered an emerging alternative in surface coating of implants due to the flexibility in tailoring them to desired mechanical and biological properties as well as owing to their low costs [20]. TKP hydrogel grafted with AA (TKP-AA) in the ratio of 1:5 has exhibited enhanced osteogenic maturation and mineralization even in the absence of any externally added growth factors [12,13]. When applied to various osteogenic and non-osteogenic cells, such as endothelial cells and neurons, TKP-AA was non-toxic, non-immunogenic, and biocompatible [13]. TKP-AA significantly enhances the expression of osteogenic genes osteopontin (OPN), osteonectin (ON), Runx-related transcription factor 2 (Runx2), and collagen type 1 (Col-1) to high levels indicating that TKP is a highly osteoinductive hydrogel [13]. This also indicated that TKP-AA can be suitable for improving osseointegration on titanium implants. Titanium with hydrogel coating had a grainy, undulating surface which is similar to the results reported in another study [12].

The Saos-2 cells on titanium unmodified surfaces showed flattened morphology, which is similar to previously reported studies [14]. The formation of clusters of multilayered cells on Ti-SLA disks was similar to the small, round Saos-2 cells that started to spread in 24 hours of culture on a titanium SLA surface in a previous study by Hempel et al. [7]. It has been previously reported that Saos-2 cells cultured on glass coverslips had a more flattened appearance [12,13]. However, when cultured on TKP-AA coated titanium disks, the Saos-2 cells were thicker and produced multiple filopodia, which helps in cell attachment and increases bone-implant contact. Filopodia-like extensions of osteoblasts on micropatterned surfaces are known to increase osteoblast adhesion and growth [21]. This indicates that the surface modifications used in this study supported osteoblast growth; however, cells took different shapes depending on the surface modifications present on the titanium disks.

Confocal imaging revealed increased cell numbers in Ti-SLA and Ti-TKP disks as compared to only titanium disks. Hempel et al. [7] also reported the rapid spreading of osteoblasts on SLA-treated titanium surfaces. Saos-2 cells were more crowded in Ti-TKP disks with many bundles of actin microfilaments, indicating high proliferation of osteoblast cells whereas in control surfaces fewer cells with thin actin microfilaments were visualized. This was further confirmed by MTT assay which showed that the cells were viable in SLA, TKP-AA surface modifications, and increased proliferation. Hence, it was observed in this study that TKP-AA-coated titanium disks promoted higher proliferation rates of osteoblasts similar to SLA. Since osteoblast organization and density influence bone differentiation [22]; hence, higher cell proliferation and density on the surface-modified titanium can be helpful in achieving better bone growth. Therefore, the first null hypothesis that there will be no significant difference in cytotoxic effect among the different titanium study groups, was rejected.

Saos-2 cells are mature osteoblasts whose basal ALP activity itself is very high [23]. Therefore, Saos-2 cells did not show any further increase in ALP activity upon any of these surface modifications. In this study, the ALP assay revealed that SLA and TKP-AA are highly biocompatible and maintained similarly high levels of ALP enzymatic activity as were observed in uncoated titanium disks. This further showed that SLA and TKP-AA surface modifications supported the differentiated state of osteoblasts.

Osteoblasts deposit calcium in the process of ossification and form calcium-rich nodules [24]. Calcium deposition (mineralization) in the osteoblasts was measured by Alizarin red staining. While SLA treatment has been relatively well reported to have better osteogenic properties than unmodified titanium disks [25], the data obtained from the present study suggests that coating the implants with TKP-AA hydrogel can give better results. The enhanced mineralization by TKP-AA can be attributed to the combined positive effects on supporting better osteoblast adhesion, proliferation, and ALP activity, besides upregulating the expression of osteogenic proteins such as collagen type 1, osteogenic genes osteopontin, osteonectin, and Runx-related transcription factor 2 [13]. Thus, the second null hypothesis of the study, which stated that there would be no significant difference in osteogenic potential among the different titanium study groups, was partially rejected.

## Conclusions

Taken together, our results confirm that surface modifications of titanium implants offer distinct advantages over unmodified titanium implants. SLA modified titanium is more advantageous than unmodified titanium. TKP-AA coating on titanium implants could be a better surface modification approach for enhanced and faster rate of osteoblast growth, bone mineralization and osseointegration. Further, in vivo studies using preclinical animal model, followed by clinical trials are needed to evaluate its practical efficacy as coating material on dental implants.
